# Structural Assessment of Interfaces in Projected Phase-Change Memory

**DOI:** 10.3390/nano12101702

**Published:** 2022-05-17

**Authors:** Valeria Bragaglia, Vara Prasad Jonnalagadda, Marilyne Sousa, Syed Ghazi Sarwat, Benedikt Kersting, Abu Sebastian

**Affiliations:** IBM Research Europe—Zurich Research Laboratory, CH-8803 Rüschlikon, Switzerland; vjo@zurich.ibm.com (V.P.J.); sou@zurich.ibm.com (M.S.); ghs@zurich.ibm.com (S.G.S.); bke@zurich.ibm.com (B.K.)

**Keywords:** in-memory computing, projected phase-change memory, interface engineering, confined phase-change material, sputtering deposition, X-ray reflectivity, STEM

## Abstract

Non-volatile memories based on phase-change materials have gained ground for applications in analog in-memory computing. Nonetheless, non-idealities inherent to the material result in device resistance variations that impair the achievable numerical precision. Projected-type phase-change memory devices reduce these non-idealities. In a projected phase-change memory, the phase-change storage mechanism is decoupled from the information retrieval process by using projection of the phase-change material’s phase configuration onto a projection liner. It has been suggested that the interface resistance between the phase-change material and the projection liner is an important parameter that dictates the efficacy of the projection. In this work, we establish a metrology framework to assess and understand the relevant structural properties of the interfaces in thin films contained in projected memory devices. Using X-ray reflectivity, X-ray diffraction and transmission electron microscopy, we investigate the quality of the interfaces and the layers’ properties. Using demonstrator examples of Sb and Sb_2_Te_3_ phase-change materials, new deposition routes as well as stack designs are proposed to enhance the phase-change material to a projection-liner interface and the robustness of material stacks in the devices.

## 1. Introduction

Chalcogenide phase-change materials (PC-materials) are well-known for their crystalline-to-amorphous transitions that are both fast and reversible, offering a large electrical and optical contrast between the two phases [1,2]. This class of materials has greatly contributed to the development of highly dense data storage in optical media and to solid-state non-volatile memory [3].

In recent years, phase-change memories (PCMs) have gained ground as non-volatile memories in the field of analog in-memory computing and neuromorphic systems [4,5,6,7]. It has been shown that PCM devices can potentially store a continuum of resistance values. When organized in crossbar arrays, PCMs can be used to perform vector-matrix multiplications, the core calculation of artificial intelligence (AI)’s inference and training tasks, by exploiting Ohm’s and Kirchoff’s laws.

However, initially developed for binary data storage [8,9] rather than for computation, the technology endures significant non-idealities due to the intrinsic material physics of PC-materials. Resistance drift and electrical read noise induce temporal variations in the device resistance [10,11], reducing the numerical precision that is achievable with this technology. 

To minimize these non-idealities, various solutions based on both materials and device engineering have been proposed [12,13,14]. Among the newly proposed cell architectures, the concept of projected PCM has gained much interest [13,15,16]. In this concept, the physical mechanism of resistance storage is decoupled from the noisy information retrieval process that is affected by the electrical properties of the PC-material’s amorphous phase.

**Figure 1:**
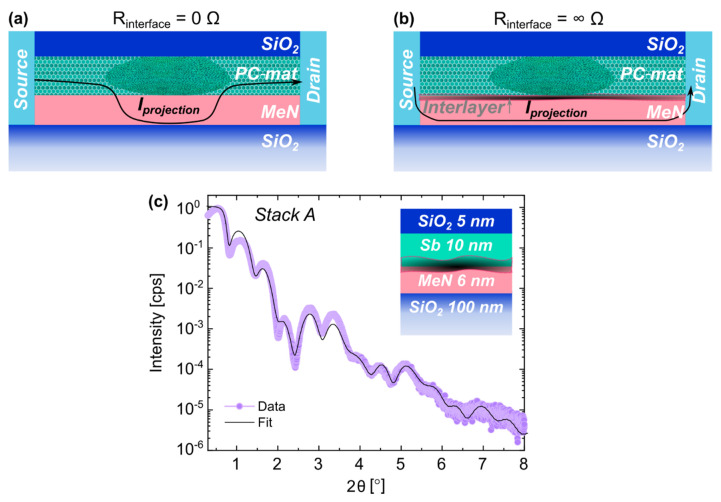
Sketch of a projected line-cell in cross sectional view with a partially amorphized PC-material layer: (**a**) in the scenario of “zero-interface resistance”, the projection current mostly bypasses the amorphous phase; (**b**) if the interface resistance is infinite, the projection current bypasses the entire phase-change layer; (**c**) XRR scan (lilac) and fit comparison (black) for the original layer stack order, *Stack A*, used in previous projected phase-change memories [18]. The sample was deposited at RT.

The decoupling is realized by using an electrically conducting material, called the projection liner (PL), placed in parallel to the PC-material, as illustrated in Figure 1a. The resistance of the PL is judiciously chosen such that it only has a marginal influence on the write operation. In effect, the device read-out characteristics become dictated by the properties of the PL. Projected phase-change memory devices have been shown to significantly reduce resistance drift and 1/f noise by at least one order of magnitude. Such improvements enable in-memory arithmetic operations with high precision [17].

Two recent studies focused on the understanding of the “projection”, investigating the device characteristics for line-cell architectures [18] as well as for vertical devices, the so called “mushroom-type phase-change memory devices” [16]. Based on these two studies, a comprehensive device model was developed to capture the behavior of the memory device for any arbitrary device state. For line-cell architectures, it was found that the interface resistance between the PC-material and PL is a crucial parameter, determining how effectively the projection works. It hampers the current flow into the PL and, thus, determines the fraction of read current that bypasses the amorphous PC-material volume [18]. As for a lateral-type projected PCM, the two extreme scenarios for R_interface_ = 0 Ω and R_interface_ = ∞ Ω are illustrated in Figure 1a,b, respectively. For a “zero-interface resistance”, the current mostly bypasses the amorphous PC-material region, leading to reduced non-idealities. If the interface resistance is infinite, the projection current bypasses the entire phase-change layer, eventually leading to a binary cell behavior. Moreover, it was shown that the interface resistance also affects both the drift characteristics and the state dependence of the device resistance. An experimental validation of the findings was also provided for projected PCM with Sb or Ge_2_Sb_2_Te_5_ material on top of a metal nitride (MeN) PL [16,18].

Here, we extend the understanding of previous studies based on a new perspective: we establish a metrology to assess relevant structural properties in projected PCM. X-ray reflectivity (XRR), X-ray diffraction (XRD), scanning transmission electron microscopy (STEM) and energy-dispersive X-ray spectroscopy (EDX) are orchestrated to determine the quality of the layer stacks with a focus on the interface and layer attributes. Spurious layers are identified and discussed in the framework of the interfacial resistance and the non-ideal device performances of previous studies [17,19]. The investigation applies to thin Sb as well as Sb_2_Te_3_ material systems for thicknesses from 10 nm down to the ultra-scaled case of 3 nm.

Several deposition approaches as well as stack variations are proposed to enhance not only the PC-material-to-PL interface but also the uniformity of the PC-material and the robustness of material stacks against thermal stress and a resulting potential mechanical strain upon fabrication. Eventually, we establish the groundwork for the conscious designing of the next generation of projected phase-change memory.

## 2. Materials and Methods

The various stacks were deposited using an FHR.Star.75.Co sputter tool. This is a multi-target sputtering system with five sputter sources, one of which was used for the inverse sputter etch process. In addition, the tool was fitted with a heating substrate stage that can reach a maximum temperature (T) of 600 °C. It can deposit multiple layers of metallic, phase-change and dielectric thin films in situ at elevated temperatures.

For this study, each layer stack was deposited in situ. This permitted a full preparation without breaking the vacuum and, therefore, prevented the oxidation and contamination of the layer stacks. The deposition parameters for the various layers are summarized in Table 1.

To analyze the structural properties of the stacks, such as phase, thickness, density and interface quality between layers, XRD and XRR measurements were performed in a Bruker D8 discover diffractometer equipped with a rotating anode generator.

The fitting analysis of the XRR data was performed using the Leptos Reflectivity software [19].

To validate the multilayer stack model obtained from the XRR and further check the chemical species present, cross-sectional lamellas were investigated. They were prepared using an FEI Helios Nanolab 450 S focused ion beam and characterized by STEM and EDX. The bright-field images were acquired using a double spherical aberration-corrected JEOL JEM-ARM200F microscope operated at 200 kV. During lamella preparation, an ion beam source with currents varying from 7 pA to 0.23 nA at 30 keV was used to thin down the lamella. Final lamella thinning was carried out using a 7 pA ion beam current at 5 keV.

The EDX line profiles were performed using a liquid-nitrogen-free silicon drift detector.

## 3. Results

The results of this study are organized into different sections.

To start off, in Section 3.1, we perform a structural assessment of a typical projected PCM layer stack order [18] based on 10 nm Sb film. We focus on the PL-to-PC-material interface quality and stability, obtained by confining thin Sb film in between different layers. Several deposition approaches as well as stack variations are proposed and compared one by one. The limit case of a 3 nm thick Sb layer is discussed in Section 3.1.1.

Subsequently, in Section 3.2, we extend the study and compare the findings to another thin PC-material, Sb_2_Te_3_.

Eventually, to highlight the advantages of the new proposed stack variations, a thermal stability test of the PL-to-PC-material interface is presented in Section 3.3 for both the Sb and Sb_2_Te_3_ case studies.

The various samples and corresponding layer stack configurations investigated in this work are listed in Table 2. The results of their structural characterizations are detailed in the following sections.

### 3.1. Projection Phase-Change Memory Stack with Sb

Figure 1c shows the XRR pattern of layer *Stack A*, as depicted in Figure 1a,b. The PC-material is deposited on the PL, and all layers are deposited at room temperature (RT). The inset of Figure 1c shows the stack schematic with the nominal thickness (not to scale).

To obtain quantitative information on the various layer densities, thickness and the interfacial roughness, the XRR curves were analyzed by fitting a simulated curve based on a multilayer model to the measured data [19]. The fit (black line) is in good agreement with the experimental data points (in lilac) in Figure 1. The outcome of the analysis is reported in Table 3.

The Sb film did not grow as a single uniform layer. An Sb layer with a lower density of 4.4 g/cm^3^ was followed by a second one with a higher density of 6.2 g/cm^3^. The latter agrees well with the density of crystalline Sb (c-Sb) found in the literature [20]. The lower density layer, instead, could be attributed to a residual amorphous onset layer (a-Sb). This was previously reported for the stabilization of thin (<10 nm) Sb and PC-materials stacked between different electrode materials [21,22,23,24].

A striking finding is the presence of an ultralow density (1.0 g/cm^3^) layer between the liner and the PC-material that we refer to as a “spurious interfacial layer”. It resulted in a poor adhesion between the Sb and the MeN, requiring a particularly careful handling of the samples during the fabrication of the actual line-cell devices.

The roughness at the various interfaces and at the surface was relatively smooth with values ≤0.3 nm (see Table 1). More details about the goodness of the fit and the significance of the extracted results are discussed in the Supplementary Materials (see Figure S1).

We propose several deposition variations to eliminate the “spurious interfacial layer” and to improve the interface stability between the different layers in the stack.

**Figure 2:**
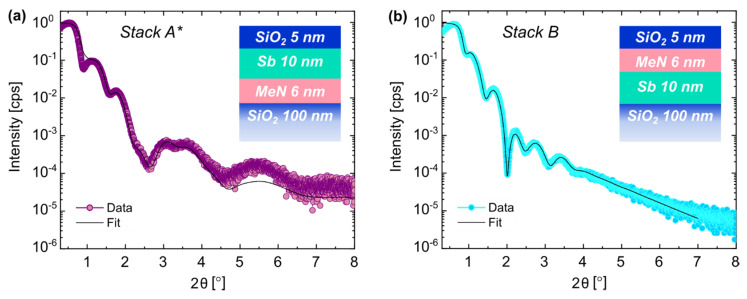
XRR scans and fit comparison for layer Stack A *, deposited at T = 200 °C (**a**), and layer *Stack B* with inverted PC-material and projection layer order, deposited at T = 200 °C (**b**).

In a first approach, the deposition of all layers was performed at an elevated temperature (T = 200 °C), as it has been shown that temperature is a critical parameter to tune the growth of thin PC-materials [22,23,24,25]. The results of the structural investigation are shown in Figure 2a and Table 4. The deposition of the PC-material at elevated T improved the PL-to-PC-material interface. However, a thin onset of a 1 nm a-Sb layer with a density of 5.0 g/cm^3^ was still needed for the fit.

The second approach was reordering the sequence of the thin films. That means the PC-material-to PL-order was inverted, as shown in the inset of Figure 2b. This path can be split into two alternatives. In one case, the layer deposition was performed at RT (see *Stack C* in the Supplementary Materials). In the other case, the layers were deposited at T = 200 °C (see Figure 2b). The advantages of inverting the order between these two layers are manifold. As shown in Table 5 and in Figure S2 of the Supplementary Materials, this approach eliminated the “spurious interfacial layer” between the PC-material and the MeN. Additionally, the deposition at elevated temperatures also improved the adhesion to the SiO_2_ substrate (Table 5 vs. Table S1). A slight increase in the interfacial and surface roughness was obtained compared to the 0.3 nm value found for the RT depositions. Nevertheless, they were still reasonably smooth (≤1 nm) (Table 5 vs. Table 3 and Table S1).

**Figure 3:**
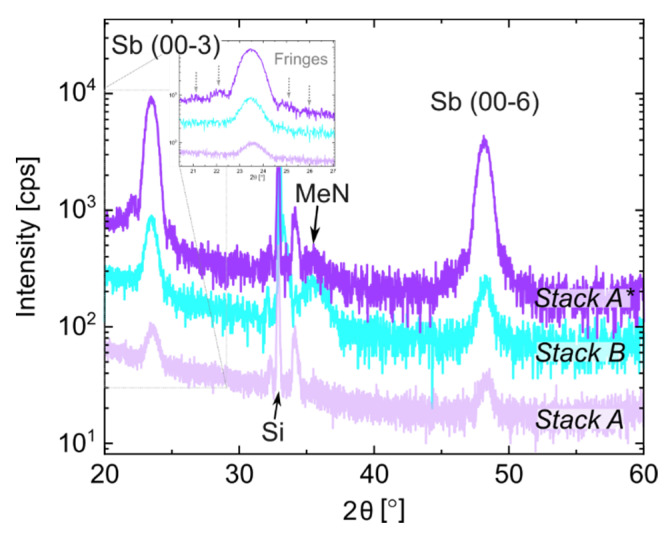
XRD profile comparison between *Stack A*, deposited at RT (lilac); Stack A *, deposited at 200 °C (purple); and *Stack B*, deposited at 200 °C with an inverted PC-material/PL layer order (cyan). The fringes around the Sb (00-3) peak are highlighted in the inset.

Next, we examined the microstructural properties of the films using XRD. We acquired and compared the XRD scans for *Stack A* (lilac scan), Stack A * (purple scan) and *Stack B* (cyan scan) in Figure 3. The peaks labeled as Sb (0003) and Sb (0006) correspond to the third and sixth order reflection of the PC-material film in the trigonal phase (R-3m space group) [26]. We made the following observations based on the characterization. There were differences in the peak areas between the traces of the three-layer stacks, which indicate that the crystalline Sb fractions in the thin films were different. It was the lowest for *Stack A* (deposited at RT) and higher for Stack A * and *Stack B*, which were both deposited at T = 200 °C. The presence of fringes in Stack A * highlights that the sample had high-quality interfaces with well-defined contrasts between the various layers in the stack. From the fringes’ periodicity around the peak Sb (00-3) of *Stack B* (see inset of Figure 3), we extracted the thickness of the crystalline Sb layer, which amounted to 8.5 nm. This value compares well with the thickness obtained using XRR (see Table 4).

The XRD profiles of the samples deposited at a high T show that the MeN layer started to crystallize. This is demonstrated by the broad band between 33° and 38° that is attributed to the MeN film. The sharp peaks in the same band are due to optics and the partial Si reflection from the substrate [27].

The deposition at elevated T and consequent crystallization of the MeN changed the resistivity of the layer with respect to the one that was intentionally chosen during the design of the device. The sheet resistance dependence of the MeN can be found in Figure S3. Considerations on the impact at the device level will follow in Section 4.

#### 3.1.1. Case Study of Ultrathin Sb

When designing a PCM cell, downscaling the thickness of the PC-material is known to be an important parameter to tune device metrics, including the resistance window, programming currents and amorphous phase retention [16,24,28]. Hence, we investigated the limit case of an ultrathin 3 nm Sb (*Stack D*).

**Figure 4:**
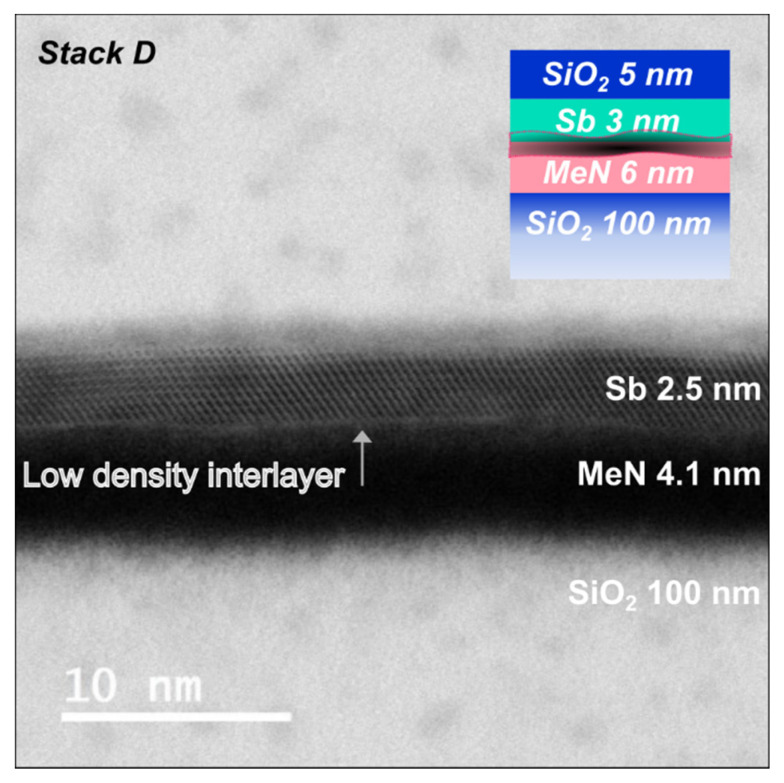
Cross-sectional bright-field STEM micrograph of *Stack D*, deposited at RT after device fabrication.

Figure 4 shows a STEM micrograph taken of a PCM cell based on *Stack D* (see schematic). Despite the layers originally being deposited at RT, the stack saw 180 °C during the fabrication of the cell.

All layers can be clearly identified, and the PC-layer was mostly crystalline. Nevertheless, the bright thin layer at the interface between Sb and MeN indicates the presence of a low-density “spurious interfacial layer”. If we compare this with the XRR analysis of the layer stack prior to device fabrication (see Table 6 and Figure S4), the results are in good agreement. Underneath the 1.7 nm crystalline Sb layer, a low-density (3.5 g/cm^3^) interlayer was found. This layer, as found in *Stack A*, was also deposited at RT but with thicker Sb (10 nm) indicates poor adhesion and could potentially lead to delamination. The slight mismatch in thickness of the crystalline layer could be attributed to the elevated T seen by the layers during fabrication or to the known slight mismatch in thickness calibration between the STEM and XRR techniques. Eventually, the RT deposition of the projected PC-layer stack with ultrathin (3 nm) Sb led to similar results as the one with 10 nm Sb.

**Figure 5:**
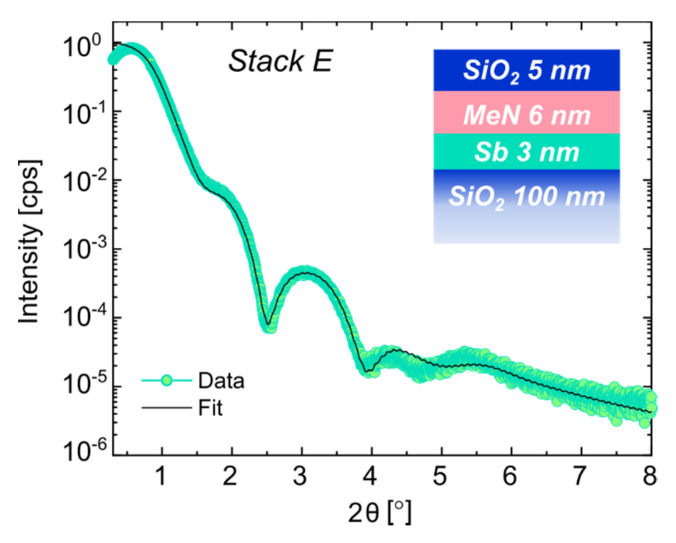
XRR scan and fit comparison for layer *Stack E*, deposited at T = 200 °C.

To further prove the advantages of the approach using elevated-T deposition and inverted PC-material and PL order, we applied it to the ultrathin 3 nm Sb case. The resulting sample was labeled as *Stack E*. The XRR profile and fit of *Stack E* are discussed based on Figure 5.

The decay of oscillations at higher angles (2θ > 4°) indicates a higher surface roughness with respect to the RT growth (see Figure 1 and Figure 5). As obtained for the similarly grown *Stack B* with a thicker 10 nm PC-layer (see Figure 2b), there was no “spurious interlayer” at the interface between the now ultrathin PC-material and the PL nor between the PC-material and the bottom SiO_2_ (see Table 7). On the contrary, the adhesion between the MeN and PC-material layers was improved, as indicated by the presence of the denser ultrathin Sb film between them. As for *Stack B*, also deposited at an elevated T, the roughness of the MeN and the SiO_2_ layers increased to values of 0.7 and 0.8 nm, respectively, due to the temperature. The Sb layer was fully crystalline, i.e., no sign of a-Sb was noted.

### 3.2. Projection Phase-Change Memory Stack with Sb_2_Te_3_

With the intent of generalizing the results on Sb-based stacks to another confined thin PC-material system, the study presented in Section 3.1 was repeated on Sb_2_Te_3_.

**Figure 6:**
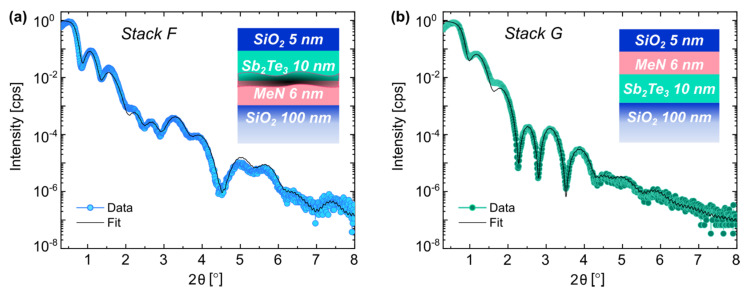
XRR scans and fit comparison for layer *Stack F*, deposited at RT (**a**), and layer *Stack G* with inverted PC-material and projection order. The sample was deposited at 200 °C (**b**).

Figure 6 shows the XRR curves of *Stack F* and *Stack G*, in blue and green, respectively. The former was deposited at RT with the original layer order, seeing the PC-material on top of the projection one. The latter was deposited at 200 °C with the inverted order of PC-layer and MeN. The results of the quantitative analysis derived from the fits (black lines) are listed in Table 8 and Table 9, respectively. As for the samples with Sb, the decay in the oscillations at high angles (2θ > 4°) for deposition at elevated T was reflected by an increased interfacial and surface roughness. A validation of the XRR surface roughness for *Stack G* is given by atomic force microscopy in Figure S5.

By growing the PC-material on top of the MeN, an ultrathin layer of ultralow density was formed between the layers, which later resulted in delamination of the samples upon handling. The density of the PC-material was low, 4.7 g/cm^3^ on average, pointing toward a mostly amorphous layer. Instead, in *Stack* *G* (Figure 6b) the Sb_2_Te_3_ had a higher density, hinting at its crystallinity [29,30]. Despite being fully crystalline, a double layer (1-Sb_2_Te_3_ and 2-Sb_2_Te_3_) was needed to account for the density variations across the layer. The reason could be attributed to the presence of different strain conditions at the top and bottom interfaces with SiO_2_ and MeN, resulting in inhomogeneous PC-material nucleation and crystallization across the layer [21,31].

**Figure 7:**
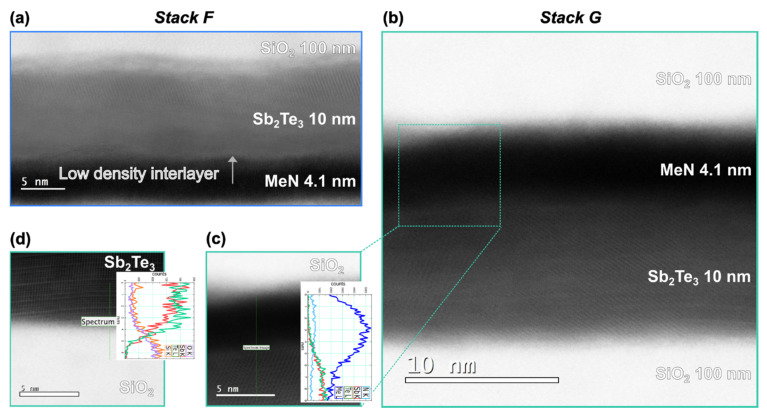
Cross-sectional bright-field STEM micrographs of *Stack F* (**a**), *Stack G* (**b**) and examples of zoomed-in images with line profiles on the MeN/Sb_2_Te_3_ interface in *Stack G* (**c**) and the Sb_2_Te_3_/SiO_2_ interface of *Stack G* (**d**).

To further validate our multilayer model of the two samples, STEM imaging was performed. The micrographs of *Stack F* and *Stack G* are shown in Figure 7a,b, respectively.

Figure 7a shows a STEM micrograph taken of a PCM cell based on *Stack F*. Despite the layers originally being deposited at RT, the stack saw an elevated T of 200 °C during the fabrication of the cell. All layers were clearly identified, and their thickness compares well with the one obtained from the XRR analysis. The Sb_2_Te_3_ layer appears predominantly crystalline, despite the lower density value (4.7 g/cm^3^) obtained for *Stack F* prior to fabrication, which implied a mostly amorphous a-Sb_2_Te_3_ layer (see Table 8). The crystallinity of the layer in Figure 7a was attributed to the elevated T of 200 °C seen during fabrication.

A bright ultrathin layer was visible at the interface between the PC-material and the MeN. It corresponds to the “spurious interfacial layer”, which was also detected in the XRR (Table 8).

The micrograph of *Stack G* shows distinct layers of thin films, and their overall thicknesses compare well with the ones obtained from the corresponding XRR (Table 9).

The Sb_2_Te_3_ layer was fully crystalline. Figure 7c is a typical zoomed-in image of the area near the PC-material /projection layer region to highlight the structural quality of this interface and the absence of any spurious low-density layers. Despite this, the EDX line scan detected a ~3 nm overlap of the metal (Me) and Sb/Te element signals across this interface. The grains of both Sb_2_Te_3_ and MeN could be responsible for this projection effect during the measurement. Moreover, Me injection into the Sb_2_Te_3_ lattice during the lamella preparation cannot be fully excluded. Moreover, in a previous study it was shown that the chemical affinity between two metals and the resulting Ti_3_Te_4_ compound formation at a higher T could explain the improved adhesion between the electrode and the PC-material [25]. Figure 7d is a zoomed-in image near the PC-material/SiO_2_ region. The interface was sharp, and no “spurious interlayer” could be identified by the STEM or by the EDX analysis, as shown in the inset.

### 3.3. Thermal Stability Study of the Interfaces with Sb and Sb_2_Te_3_

**Figure 8:**
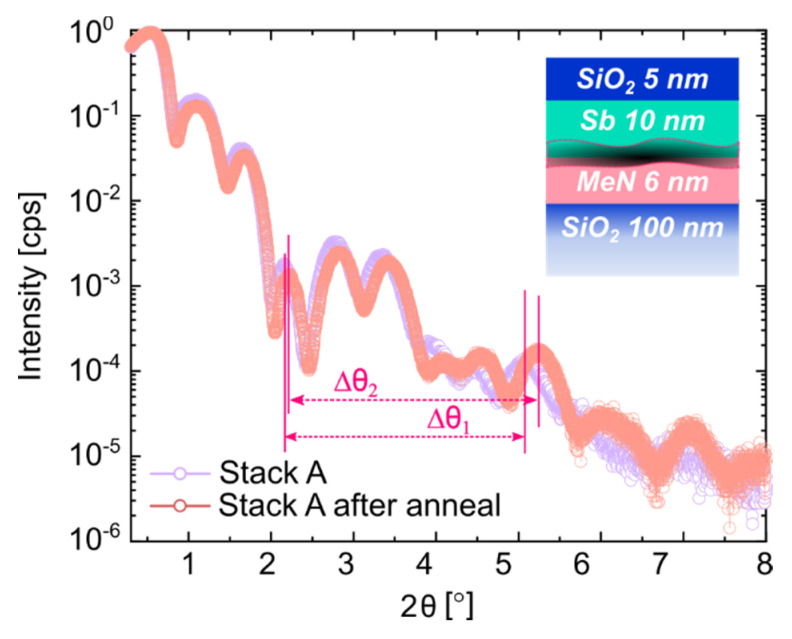
XRR scan comparison for layer *Stack A*, as deposited at RT (lilac), and after annealing at 220 °C (orange).

To highlight the advantages of the newly proposed stack variation, a thermal stability test of the PL-to-PC-material interface is presented for both the Sb and the Sb_2_Te_3_ case studies. Figure 8 compares the XRR patterns of the RT-deposited *Stack A* (colored in lilac), with Sb as PC-material, and the one obtained after annealing at T = 220 °C for 30 min (in orange). The periods of the Pendellösung interference fringes are labeled as Δθ_1_ and Δθ_2_ for *Stack A* before and after annealing, respectively. The oscillations of *Stack A* before annealing (in lilac) display a slightly shorter oscillation period (Δθ_1_ = 2.9° < Δθ_2_ = 3.0°). Hence, a change in film thickness for one or more layers occurred upon annealing [32,33]. For larger scan angles (2θ > 4°), the differences in the oscillation profile variations become more evident, highlighting major changes in the deeper layers of the stack. The curves showed no relevant decay of oscillation amplitude, suggesting that the interface roughness did not increase significantly. A change in surface roughness can be disregarded as well, as no fast decay of the XRR signal was detectable [33].

The result of the quantitative analysis of *Stack A* after annealing, obtained by fitting the measured data, is reported in Table 10 and confirms the assumptions based on the XRR profile comparisons. A slight increase (~0.7 nm) in the crystalline portion of Sb was detected compared to the sample before annealing, with an accompanying decrease in the amorphous layer contribution of ~1 nm (see Table 3 for comparison). The critical role of the “spurious interlayer” increased upon annealing at an elevated T, as demonstrated by its even lower density value of 0.3 g/cm^3^ compared to 1.0 g/cm^3^ for *Stack A* before annealing. This later resulted in a delaminated sample upon handling (not shown). See Table 3 and Table 10 for comparisons.

**Figure 9:**
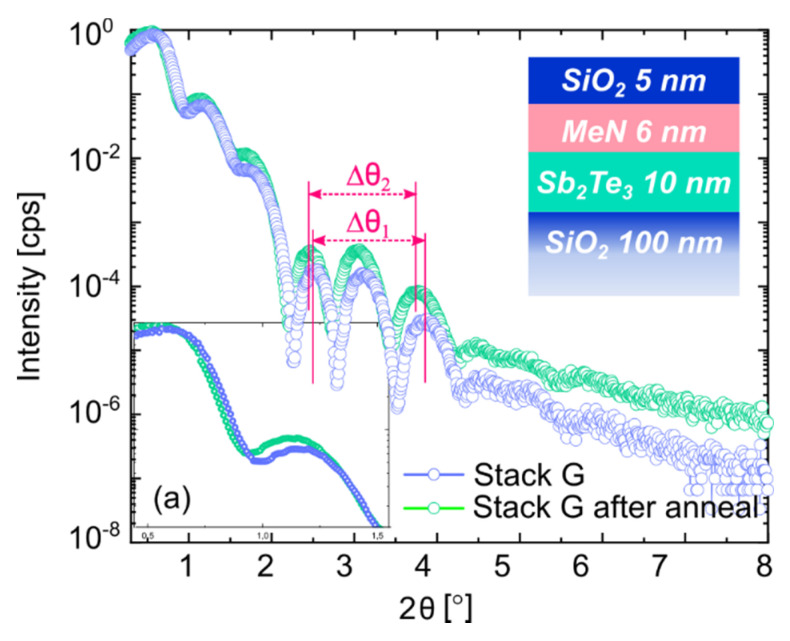
XRR scan comparison for layer *Stack G*, as deposited at 200 °C (blue) and after annealing at 220 °C (green). The Pendellösung fringes and their periodicity are marked with Δθ_1_ and Δθ_2_ (in red). (a) The inset shows a zoomed-in image of the total reflection edge.

The same temperature study on the Sb_2_Te_3_-based *Stack G*, with inverted PC-material and PL order, led to different results. Figure 9 compares the XRR profiles of *Stack G* before and after annealing at T = 220 °C for 30 min.

In this case, the two curves show more pronounced differences. The total reflection edge is enlarged for the sake of clarity and shown in the inset (a) of Figure 9. The edge of the annealed film shifted toward smaller angles, indicating that one or more layers had a lower density with respect to the case before annealing [32,33]. The periods of the interference fringes are labeled as Δθ_1_ and Δθ_2_ for *Stack G* before and after annealing, respectively. From their comparison, information about the thickness change upon annealing can be extracted [33].

In particular, the oscillations of *Stack G* after annealing (green data points) displayed a shorter oscillation period (Δθ_2_ = 1.3° < Δθ_1_ = 1.4°) compared to before annealing (blue data points). Thus, an increase in film thickness occurred upon annealing. These observations were confirmed by the quantitative analysis and the change in the roughness, thickness and density of *Stack G* after annealing, as reported in Table 11 (see Table 9 for comparisons).

The temperature treatment converted the double Sb_2_Te_3_ layer into a uniform 8.3 nm layer of Sb_2_Te_3_ with a density of 5.0 g/cm^3^. This value was lower compared to the ones reported in Table 9.

No ultralow-density “spurious interlayer” that was responsible for adhesion issues was detected at the interface with the SiO_2_ or the interface with the projection layer, as desired. The curves show no relevant decay of the oscillation amplitude, suggesting that the interface roughness did not change significantly. Nevertheless, the slight decrease in the top surface layer roughness could be responsible for the higher XRR signal for *Stack G* after annealing.

## 4. Discussion

The results shown in Section 3 demonstrate how critical it is to control the layer stack deposition processes prior to projected phase-change memory fabrication. To fine-tune the layer and interfacial properties of the various materials in the stack, a solid methodology to characterize and model the multilayer stack is paramount.

Based on XRR, XRD, STEM and EDX, we have observed that by depositing the PC- material on the PL at RT, as performed in a previous study [18], a spurious interlayer is formed between the two layers. As a result of the ultralow density of the spurious layer, adhesion issues and, eventually, delamination can occur upon sample manipulation. In Kersting et al. [18], it was shown that in the limiting case of infinite interface resistance, the PCM cell could lose the multistate resistance property and eventually act as binary memory by completely bypassing the presence of the PL. The existence of such a “spurious layer” in the samples *Stack A*, *Stack D* and *Stack F* results in a non-ideal contact resistance between the PC-material and the MeN. We assume it to be a contributing factor, if not the main cause, of the non-ideal projection properties observed in the phase-change memory cells. These stacks lead to cells described by the scenario in Figure 1b.

**Figure 10:**
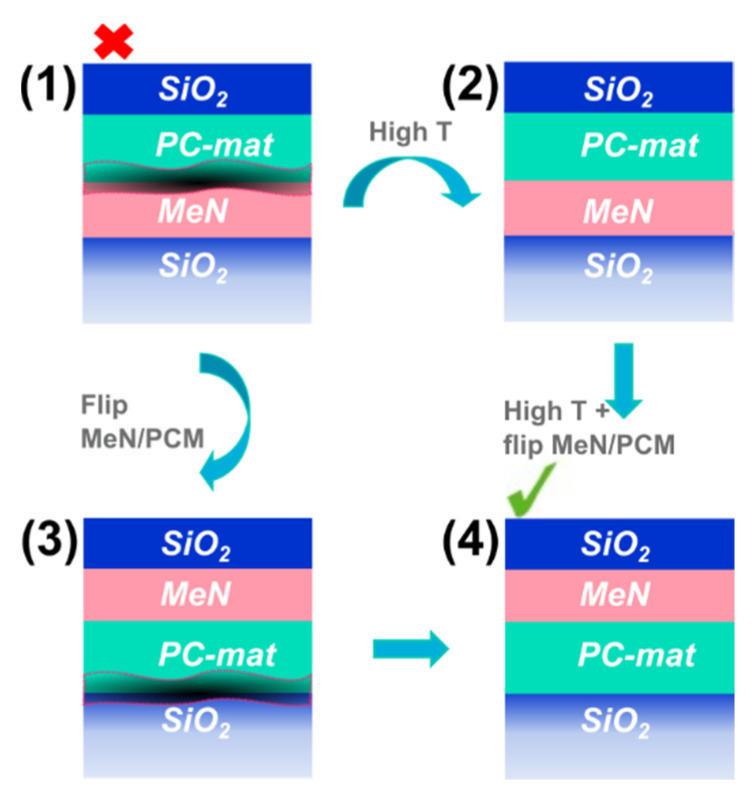
Summary of the four major stack deposition approaches pursued to enhance the PC-material-to-MeN interface and the uniformity of the PC-material.

Figure 10 shows an overview of the deposition approaches we proposed as well as stack variations to enhance the PC-material-to-PL interface and the uniformity of the PC-material.

(1) is the schematic of the original stack used for previous studies [18] that shows the spurious interface and results in easy delamination. In (2), the same layer order is kept, but the full stack is deposited at 200 °C. This leads to an improved PC-material-to-MeN interface, but nevertheless, the PC-material is not very uniform and, once processed into line-cell devices, it results in a binary behavior of the memory (not shown). In (3) the order between PC-material and MeN is inverted, but the full layer stack is deposited at RT. The advantage in this case is that there is no spurious layer between the PC-material and MeN, which is desirable. Nonetheless, a low-density interlayer is found between the PC-material and the bottom SiO_2_ layer. The most robust approach is shown in (4). It combines approaches (2) and (3), yielding a sharp PC-material-to-projection-layer interface and a more uniform phase of the PC-layer. The adhesion of the PC-material to the bottom SiO_2_ is also improved.

One more advantage of approach (4) is the larger freedom in designing the phase-change memory by decoupling the PC material deposition from that of the PL. The PC-material layer could be deposited at elevated T to improve the adhesion with the layer underneath and its phase uniformity. The PL on top could then be deposited at RT or any other T to consciously tune its resistivity. This precaution is important since properties such as the resistance of the PL have been shown to change with T (see Figure S2), leading to projected PCM devices that unintentionally differ from their original designs.

## 5. Conclusions

Projected PCM devices are an unfolding path toward the implementation of high-precision in-memory computing. However, to exploit their full potential, the origin of the interfacial resistance must be understood and controlled.

In this work, a dedicated structural investigation of the memory stacks using XRD, XRR, STEM and EDX revealed the presence of a spurious ultralow-density layer between the PC-material and the projection liner, which resulted in poor adhesion. This layer impaired the contact resistance between the PC-material and PL, leading to the suboptimal projection properties observed in the PCM cells.

To fine-tune the layers and interfacial properties of the various materials as well as to increase their stability in the memory cells, alternative deposition routes and stack designs were proposed and analyzed one by one.

We also established a schematic framework for designing projected phase-change memories for analog in-memory computing by advancing both the understanding and the tailoring of the interfacial resistance. The learning also applies to other applications where PCM devices are based on heterostructures.

## Figures and Tables

**Table 1:** Summary of the deposition parameters used for the various samples’ layers.

Layer	Deposition Parameters
MeN	Reactive sputtering
Sb	DC sputtering: 50 W, 3 µbar, 110 sccm Ar
Sb_2_Te_3_	DC sputtering: 60 W, 6 µbar, 110 sccm Ar
SiO_2_ capping	RF sputtering: 250 W, 6 µbar, 110 sccm Ar

**Table 2:** Overview of the samples investigated in this study. The layer order is listed from top to bottom.

Sample ID	Layer Stack Order (Top to Bottom)	Deposition T
*Stack A*	SiO_2_/10 nm Sb/6 nm MeN/SiO_2_	RT
Stack A *	SiO_2_/10 nm Sb/6 nm MeN/SiO_2_	200 °C
*Stack B*	SiO_2_/6 nm MeN/10 nm Sb/SiO_2_	200 °C
*Stack C*	SiO_2_/6 nm MeN/10 nm Sb/SiO_2_	RT
*Stack D*	SiO_2_/3 nm Sb/6 nm MeN/SiO_2_	RT
*Stack E*	SiO_2_/6 nm MeN/3 nm Sb/SiO_2_	200 °C
*Stack F*	SiO_2_/10 nm Sb_2_Te_3_/6 nm MeN/SiO_2_	RT
*Stack G*	SiO_2_/6 nm MeN/10 nm Sb_2_Te_3_/SiO_2_	200 °C

**Table 3:** Output of the XRR fit analysis for *Stack A*.

*Stack A*	Thickness (nm)	Roughness (nm)	Density (g/cm^3^)
SiO_2_	5.6	0.3	2.3
c-Sb	5.2	0.3	6.2
a-Sb	4.4	0.1	4.5
Interlayer	0.8	0.3	1.0
MeN	4.4	0.3	13.5
SiO_2_	100	0.3	2.4

**Table 4:** Output of the XRR fit analysis for Stack A *.

Stack A *	Thickness (nm)	Roughness (nm)	Density (g/cm^3^)
SiO_2_	5.3	1.0	2.2
c-Sb	8.4	0.7	5.6
a-Sb	1.0	0.5	5.0
MeN	4.2	0.3	13.8
SiO_2_	100	0.1	2.4

**Table 5:** Output of the XRR fit analysis for *Stack B*.

*Stack B*	Thickness (nm)	Roughness (nm)	Density (g/cm^3^)
SiO_2_	5.0	1.0	2.3
MeN	4.2	0.9	14.5
c-Sb	9.2	0.9	5.7
Interlayer	0.9	0.3	8.6
SiO_2_	100	0.1	2.4

**Table 6:** Output of the XRR fit analysis for *Stack D* prior to PCM fabrication.

*Stack D*	Thickness (nm)	Roughness (nm)	Density (g/cm^3^)
SiO_2_	5.3	0.9	2.5
c-Sb	1.7	0.4	7.0
Interlayer	1.8	0.7	3.5
MeN	4.3	0.3	14.4
SiO_2_	100	0.1	2.4

**Table 7:** Output of the XRR fit analysis for *Stack E*.

*Stack E*	Thickness (nm)	Roughness (nm)	Density (g/cm^3^)
SiO_2_	4.6	0.8	2.0
MeN	4.9	0.7	14.4
c-Sb	2.4	0.5	6.2
Interlayer	0.5	0.1	8.1
SiO_2_	100	0.1	2.4

**Table 8:** Output of the XRR fit analysis for *Stack F*.

*Stack F*	Thickness (nm)	Roughness (nm)	Density (g/cm^3^)
SiO_2_	7.2	1.0	1.5
a-Sb_2_Te_3_	10	0.5	4.7
Interlayer	0.8	0.6	0.9
MeN	4	0.4	11.0
SiO_2_	100	0.5	2.4

**Table 9:** Output of the XRR fit analysis for *Stack G*.

*Stack G*	Thickness (nm)	Roughness (nm)	Density (g/cm^3^)
SiO_2_	6.1	0.7	1.0
MeN	3.9	0.8	12.3
2-Sb_2_Te_3_	4.9	0.7	5.7
1-Sb_2_Te_3_	3.9	0.6	6.3
SiO_2_	100	0.3	2.4

**Table 10:** Output of the XRR fit analysis for *Stack A* after annealing at 220 °C.

*Stack A* (After Anneal.)	Thickness (nm)	Roughness (nm)	Density (g/cm^3^)
SiO_2_	5.7	0.1	1.9
c-Sb	5.9	0.1	6.3
a-Sb	3.3	0.3	4.6
Interlayer	1.1	0.1	0.3
MeN	4.2	0.3	12.6
SiO_2_	100	0.1	2.4

**Table 11:** Output of the XRR fit analysis for *Stack G* after annealing at 220 °C.

*Stack G* (After Anneal.)	Thickness (nm)	Roughness (nm)	Density (g/cm^3^)
SiO_2_	5.1	0.6	1.0
MeN	3.8	0.6	12.3
2-Sb_2_Te_3_	8.3	0.5	5.0
1-Sb_2_Te_3_	0.9	0.5	5.9
SiO_2_	100	0.2	2.4

## Data Availability

The data that support the findings of this study are available from the corresponding author upon reasonable request.

## Supplementary Materials

The following are available online at https://www.mdpi.com/article/10.3390/nano12101702/s1. Figure S1: XRR scan (lilac dots) and various fit comparisons for layer *Stack A*, deposited at RT; Figure S2: XRR scan (black) and fit comparison (red) for layer *Stack C*, deposited at RT and with inverted PC material/PL order. The various roughnesses, thicknesses and densities extracted from the fit are reported in the corresponding Table S1; Table S1: Output of the XRR fit analysis for *Stack C*; Figure S3: Temperature dependence of sheet resistance for MeN deposited with different nitrogen concentrations. Red data points refer to a RT deposition and green data points refer to a deposition performed at 200 °C; Figure S4: XRR scans and fit comparison for layer *Stack D*, deposited at RT; Figure S5: AFM image of a 5 × 5 µm^2^ region on *Stack G*. The values of the measured roughness (Rq) are reported on the side.
