# Supplementary material for: Structural Assessment of Interfaces in Projected Phase-Change Memory
Source: Nanomaterials (Basel). 2022 May 17;12(10):1702. doi: 10.3390/nano12101702 (PMC9147056; doi:10.3390/nano12101702)
Supplement: Supplementary file 1 [file nanomaterials-12-01702-s001.zip › nanomaterials-1691708-supplementary.pdf]

nanomaterials

Supplementary Information

Structural Assessment of Interfaces in Projected Phase-Change Memory

Valeria Bragaglia[1*], Vara Prasad Jonnalagadda[1], Marilyne Sousa[1], Syed Ghazi Sarwat[1], Benedikt Kersting[1] and Abu Sebastian[1*]

[1] IBM Research Europe - Zurich Research Laboratory, CH-8803 Rüschlikon, Switzerland.
* Correspondence: vbr@zurich.ibm.com; ase@zurich.ibm.com

Examples of fit to data agreement goodness for Stack A

1) Using two Sb_2Te_3 layers of 5.2+4.4 nm with distinct densities (see the model used in the manuscript) and densities, as well as with 0.8 nm of spurious interlayer. Model reported in the manuscript (here BLACK LINE)
2) Only using 1 Sb_2Te_3 layer of 10.4 nm with ϱ=5.4 g/cm^3 (average density between the ones of the two layers of case 1)) and no spurious interlayer (RED LINE)
3) varying the 1 layer Sb_2Te_3 thickness from 10.4 to 10 nm (BLUE LINE)

The multi stack model that allows to obtain the closest reproduction of the oscillation amplitude and period is based on 1). This model reflects a more realistic growth of ultrathin (<10nm) Phase change- films in presence of strain at the different interfaces with the substrate and electrodes, as also found in previous studies on similar material systems (see Ref 21-24 in the manuscript).

Stack A

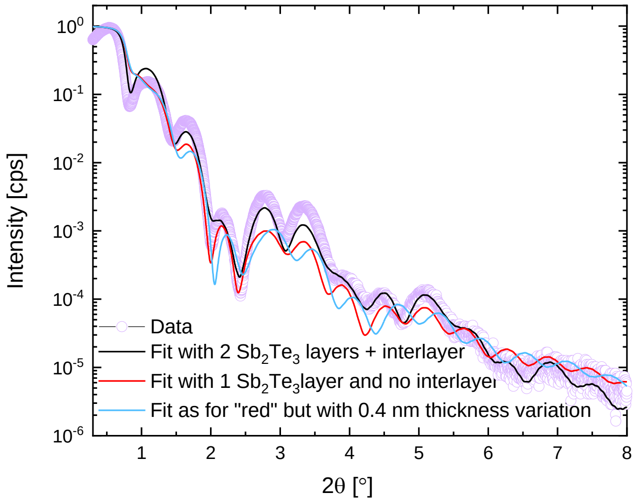

Figure S1: XRR scan (lilac dots) and various fit comparisons for layer *Stack A* deposited at RT.

Experimental data and fitting results for Stack C

Depositing the layer *Stack C* with inverted PC-material to MeN order at RT improves the interface between liner and Sb, nevertheless an ultralow density interlayer of 1.7 g/cm³ is detected between the Sb and the SiO_2 layer. This spurious layer causes poor adhesion and potentially results in delamination upon handling of the stack for fabrication.

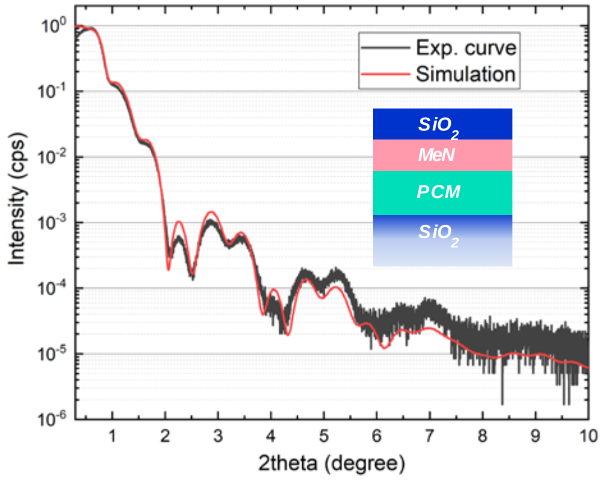

Figure S2: XRR scan (black) and fit comparison (red) for layer *Stack C* deposited at RT and with inverted PC-material/PL order. The various roughness, thickness and densities extracted from the fit are reported in the corresponding **Table S1**.

Table S1: Output of the XRR fit analysis for Stack C.

Layer stack	Thick. (nm)	Roughness (nm)	Density (g/cm³)
SiO_2	5.2	0.7	2.4
MeN	4.4	0.01	13.8
Sb	9.9	0.3	5.5
int-Sb	1.1	0.3	1.7
SiO_2	100	0.1	2.4

Temperature dependance of sheet resistance for MeN

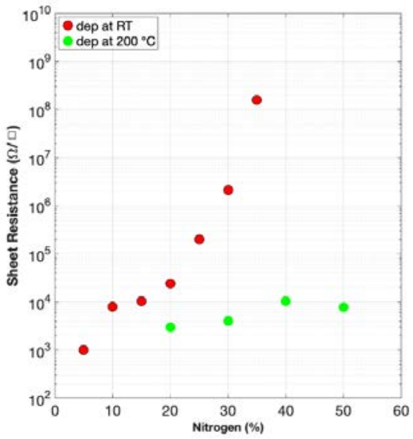

Figure S3: Temperature dependance of sheet resistance for MeN deposited with different nitrogen concentration. Red data points refer to a RT deposition and green data points refer to a deposition performed at 200 °C.

Data and fit of Stack D

The values extracted from the fit are summarized in Table 6 in the manuscript.

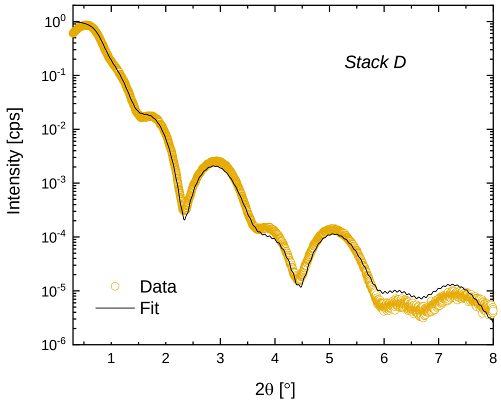

Figure S4: XRR scans and fit comparison for layer *Stack D* deposited at RT.

Validation of the XRR surface roughness for Stack G by atomic force microscopy (AFM)

We performed AFM of Stack G "as deposited" at 200°C and measured its roughness (RMS). The value of roughness obtained by XRR analysis (RMS = 0.7 nm in Table 9 of the manuscript) agrees well with the one gained by AFM (in the image below RMS = Rq = 0.689 nm).

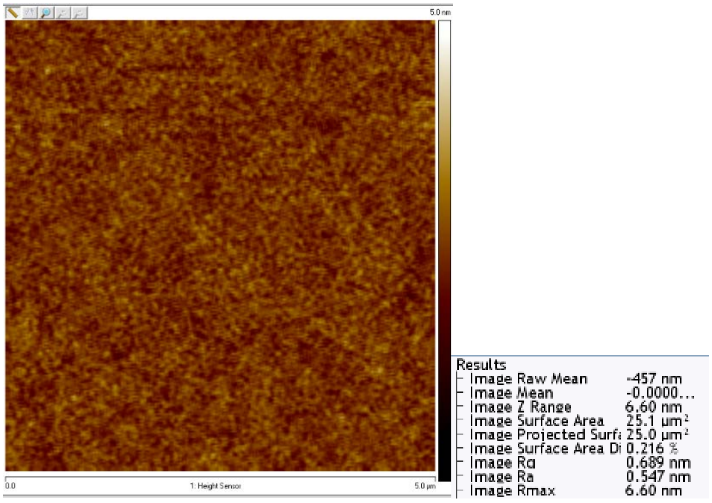

Figure S5: AFM image of a 5 x 5 μm^2 region on Stack G. The values of the measured roughness (Rq) are reported on the side
